# Psychometric revalidation of the SPRINT-E scale for assessing post-traumatic stress in Latin American populations during the COVID-19 pandemic

**DOI:** 10.3389/fpsyg.2026.1781414

**Published:** 2026-04-08

**Authors:** Christian R. Mejia, Renzo Felipe Carranza Esteban, Oscar Mamani-Benito, Víctor Serna-Alarcón, Neal M. Davies, Jaime A. Yáñez, Leonel Vega Perez, Camilo Vega-Useche

**Affiliations:** 1 Asociación Médica de Investigación y Servicios en Salud, Lima, Peru; 2 Universidad San Ignacio de Loyola, Lima, Peru; 3 Universidad Señor de Sipán, Chiclayo, Peru; 4 Universidad Privada Antenor Orrego, Piura, Peru; 5 Hospital de la Amistad Perú Corea Santa Rosa II-2, Piura, Peru; 6 Faculty of Pharmacy and Pharmaceutical Sciences, University of Alberta, Edmonton, AB, Canada; 7 Escuela de Posgrado, Universidad Internacional Iberoamericana, Campeche, Mexico; 8 Universidad Pedagógica y Tecnológica de Colombia, Tunja, Colombia; 9 Fundación Santa Fe de Bogotá, Bogotá, Colombia

**Keywords:** COVID-19, cross-national study, Latin America, mental health, post-traumatic stress disorder

## Abstract

**Introduction:**

Post-traumatic stress disorder (PTSD) has been one of the major psychological consequences observed during and after the COVID-19 pandemic. Therefore, it is essential to have valid and reliable instruments for its assessment.

**Objective:**

To evaluate the internal structure and reliability of a brief SPRINT-E-based measure for screening post-traumatic stress symptoms in Latin American populations during the early phase of the COVID-19 pandemic.

**Methods:**

This was an instrumental study using the Short Posttraumatic Stress Disorder Rating Interview (SPRINT-E) scale, administered to more than 6,000 participants across 12 Latin American countries. Descriptive statistics and confirmatory factor analysis (CFA) were conducted to evaluate the factorial structure and internal consistency of the instrument.

**Results:**

Item 4 showed the highest mean score (M = 0.85) and the greatest dispersion (SD = 0.78). The CFA supported the original unidimensional 12-item model, which demonstrated satisfactory goodness-of-fit indices. The PTSD-COVID-19 scale showed a Cronbach’s *α* of 0.92 (95% CI: 0.91–0.92), indicating excellent internal consistency.

**Conclusion:**

The PTSD-COVID-19 scale showed acceptable evidence of internal structure and excellent internal consistency as a brief instrument for screening post-traumatic stress symptoms in Latin American adults during the early phase of the COVID-19 pandemic.

## Introduction

Post-traumatic stress disorder (PTSD) is a trauma- and stressor-related disorder that, according to DSM-5 and DSM-5-TR, requires exposure to a qualifying traumatic event, a specific symptom configuration, clinically significant distress or functional impairment, and persistence of symptoms for more than 1 month (American Psychiatric Association, 2022). Compared with previous diagnostic formulations, DSM-5 refines Criterion A, separates avoidance symptoms from negative alterations in cognition and mood, and emphasizes the link between symptoms and traumatic exposure, with important implications for conceptualization and psychometric assessment (American Psychiatric Association, 2022; Weathers et al., 2014). Therefore, instruments designed to assess PTSD should not be interpreted as generic measures of anxiety or nonspecific psychological distress, but rather as tools intended to capture trauma-related symptomatology within a defined diagnostic framework (American Psychiatric Association, 2022; Weathers et al., 2014).

During the COVID-19 pandemic, high levels of anxiety, uncertainty, grief, and psychological distress are widely documented across populations; however, the assessment of PTSD in this context poses important methodological challenges (Husky et al., 2021). Recent literature emphasizes that many studies conducted during the pandemic rely on self-report symptom scales, do not adequately establish Criterion A exposure, and frequently use cross-sectional online surveys to infer PTSD prevalence (Husky et al., 2021; Norrholm et al., 2021). This distinction is critical because pandemic-related adversity is undoubtedly stressful, yet it does not universally meet DSM-5 criteria for traumatic exposure (Brunet et al., 2022; Norrholm et al., 2021). Accordingly, it is methodologically more appropriate to distinguish general psychological distress and acute stress reactions from clinically confirmed PTSD, particularly in studies conducted during the early phases of the pandemic and in general population samples (Brunet et al., 2022; Husky et al., 2021; Norrholm et al., 2021).

This distinction is especially relevant for the present study because data were collected between June and August 2020, corresponding to an early stage of the pandemic in Latin America. Consequently, the findings should be interpreted as reflecting early post-traumatic symptomatology associated with pandemic-related experiences rather than longitudinally confirmed chronic PTSD. From a measurement perspective, several instruments are used to assess trauma-related symptoms in Spanish-speaking populations. The PTSD Checklist for DSM-5 (PCL-5) is one of the most widely used instruments and has shown psychometric adaptation in Mexican samples, as well as subsequent validation, including abbreviated versions, in disaster-exposed populations (Durón Figueroa et al., 2019; Martínez-Levy et al., 2021). More recently, the International Trauma Questionnaire (ITQ) has shown acceptable psychometric evidence in Chilean adults for the assessment of PTSD and complex PTSD according to ICD-11 (Fresno et al., 2023). Nevertheless, the SPRINT-E has already been psychometrically evaluated in a Spanish-speaking Latin American sample affected by a large-scale disaster in Chile, providing a relevant regional precedent for the use of a brief trauma-related screening instrument (Leiva-Bianchi and Gallardo, 2013). On this basis, the aim of the present study is to re-evaluate a brief SPRINT-E-based measure for screening post-traumatic stress symptoms associated with COVID-19 in Latin American adults during the early phase of the pandemic.

## Materials and methods

### Design and population

An instrumental, cross-sectional study was conducted in 12 Latin American countries: Peru, Chile, Paraguay, Mexico, Colombia, Bolivia, Panama, Ecuador, Costa Rica, El Salvador, Honduras, and Guatemala. Participants were adults aged 18 years or older, residing in one of these countries, and who provided electronic informed consent. Surveys that were incomplete or inadequately filled out were excluded.

A non-probabilistic convenience sampling approach was used to recruit a large and diverse population across the region. Most respondents were from Peru, as this country served as the primary research hub and was among the most affected globally during the data collection period. Participants were recruited between 01/06/2020 and 31/08/2020 through an online self-administered survey distributed across the participating countries during the COVID-19 pandemic.

### Instrument

The instrument used in this study was a COVID-19-contextualized version based on the conceptual framework of the SPRINT-E previously evaluated by Leiva-Bianchi and Gallardo-Cuadra in a Chilean population exposed to the 2010 earthquake and tsunami (Leiva-Bianchi and Gallardo, 2013). The original SPRINT-E served as the conceptual starting point for the development of a brief instrument intended to assess self-reported post-traumatic stress symptoms in the context of the COVID-19 pandemic. Based on this framework, the research team drafted a 12-item version whose wording was subsequently refined through expert review. The final version preserved the main conceptual domains of the original brief instrument while adapting item content to the pandemic context. Each item was rated on a 5-point Likert scale ranging from strongly disagree to strongly agree.

### Procedure

The research protocol was reviewed and approved by the Research Bioethics Committee of the Universidad Privada Antenor Orrego (UPAO) (Resolution No. 0240-2020-UPAO, approved on 05/06/2020). All procedures were conducted in accordance with the Declaration of Helsinki. Before accessing the questionnaire, participants were informed about the study objectives, the voluntary and anonymous nature of participation, and the confidentiality of their responses, and they provided electronic informed consent.

First, the PTSD-COVID-19 scale was reviewed by the research team. Second, content validity was assessed by a panel of 15 experts (3 from Ecuador, 2 from Mexico, 2 from Peru, 2 from Costa Rica, 2 from Bolivia, and one each from Paraguay, Venezuela, Panama, and Colombia), who evaluated the relevance, representativeness, and clarity of each item (Ventura-León, 2019). Third, the finalized version of the PTSD-COVID-19 scale was distributed as an online self-administered survey between 01/06/2020 and 31/08/2020. Participants were recruited through non-probabilistic convenience sampling using digital dissemination by the research team and collaborators across the participating countries. The survey link was shared through online communication channels, including social media, messaging applications, and institutional or interpersonal digital networks. Participation was voluntary, anonymous, and entirely remote.

### Data analysis

Before conducting the statistical analyses, the dataset was reviewed for completeness and response quality. Incomplete or inadequately completed questionnaires were excluded. Because cases with missing item-level information were removed during this initial cleaning stage, the subsequent analyses were performed on complete cases only, and no imputation procedures were applied.

Data analysis was conducted in three phases. In the first phase, descriptive statistics (mean, standard deviation, skewness, and kurtosis) were computed using FACTOR Analysis version 10. Values greater than ±1.5 for skewness and kurtosis were considered indicators of non-normality (Pérez and Medrano, 2010).

In the second phase, a Confirmatory Factor Analysis (CFA) was performed using AMOS version 21, applying structural equation modeling (SEM) to assess model fit. The following goodness-of-fit indices were used: Comparative Fit Index (CFI), Tucker–Lewis Index (TLI), Goodness-of-Fit Index (GFI), Adjusted Goodness-of-Fit Index (AGFI), Root Mean Square Error of Approximation (RMSEA), and Root Mean Square Residual (RMR). According to Hooper et al. (2008), satisfactory model fit is indicated by CFI, TLI, GFI, and AGFI values greater than 0.90, and RMSEA values ≤0.08.

In the third phase, reliability was assessed using SPSS version 23.0 by calculating Cronbach’s alpha coefficient and corresponding confidence intervals (Domínguez-Lara, 2016) to estimate the internal consistency of the construct.

## Results

A total of 6,177 participants were included in the analytic sample. Of these, 3,705 (60.0%) were female, 2,444 (39.6%) were male, and 28 (0.5%) had missing or unspecified sex data. Table 1 presents the mean, standard deviation, skewness, and kurtosis values for the 12 items of the PTSD-COVID-19 Scale. Item 4 recorded the highest mean score (M = 0.85) and the greatest variability (SD = 0.78). The skewness and kurtosis values for most items were within the acceptable range (±1.5) (Pérez and Medrano, 2010), indicating adequate distributional properties, except for item 12, which slightly exceeded this threshold. Supplementary Table S1 presents item-level means and standard deviations stratified by country and sex.

**Table 1:** Mean, standard deviation, asymmetry, and kurtosis.

Item	M	SD	Asymmetry	Kurtosis
Item 1	0.566	0.690	0.814	−0.550
Item 2	0.633	0.706	0.656	−0.780
Item 3	0.661	0.729	0.621	−0.898
Item 4	0.853	0.784	0.265	−1.332
Item 5	0.742	0.720	0.430	−0.994
Item 6	0.589	0.692	0.752	−0.632
Item 7	0.601	0.751	0.802	−0.794
Item 8	0.785	0.743	0.368	−1.119
Item 9	0.727	0.744	0.487	−1.060
Item 10	0.732	0.733	0.465	−1.030
Item 11	0.649	0.707	0.620	−0.816
Item 12	0.202	0.483	2.381	4.902

M, mean; SD, standard deviation. Asymmetry and kurtosis values are reported to assess distributional properties of the items.

### Confirmatory factor analysis

**Figure 1:**
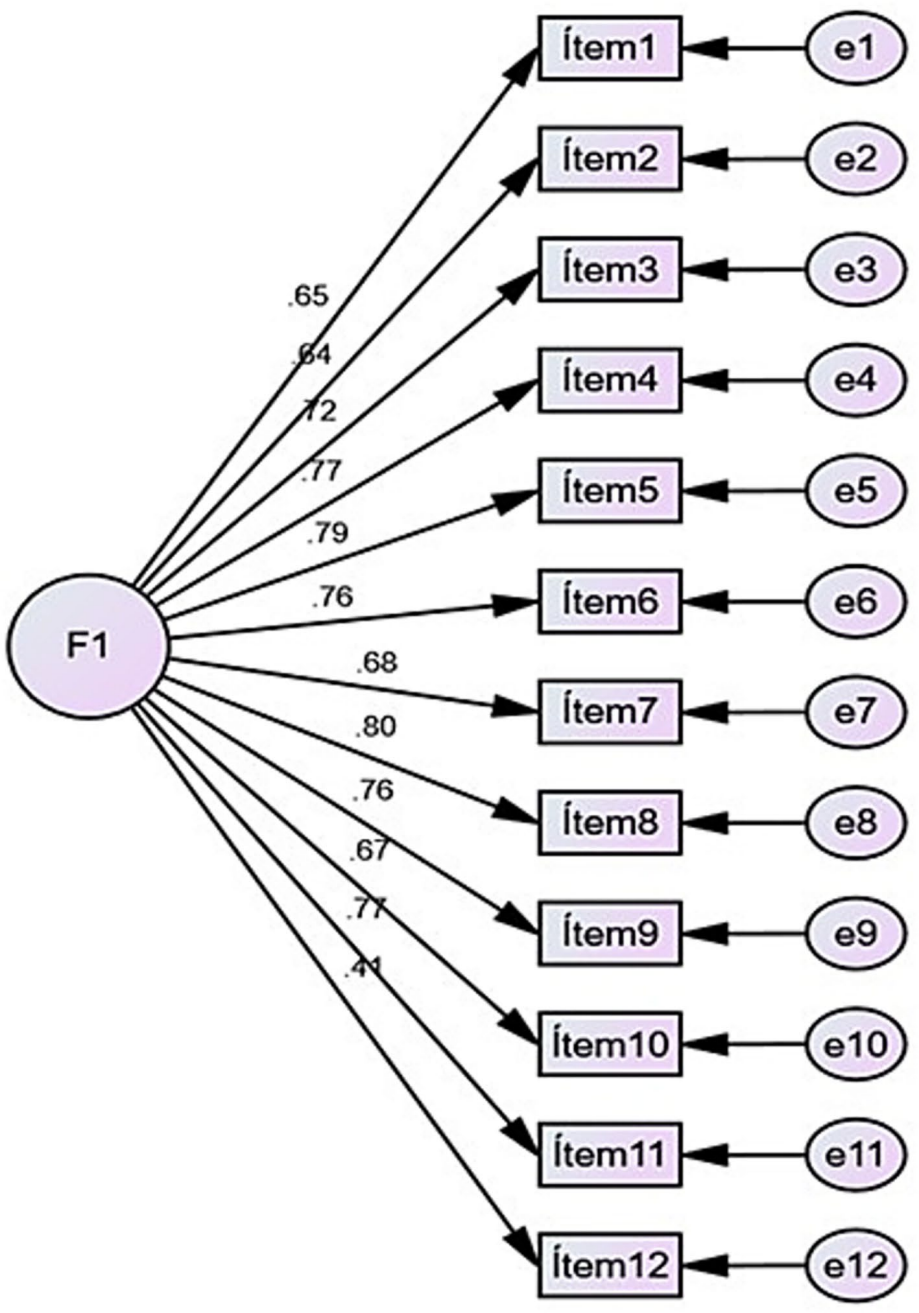
One-dimensional model of the PTSD-COVID-19 scale.

To examine the internal structure of the scale, a Confirmatory Factor Analysis (CFA) was conducted based on the original one-dimensional model, in which all 12 items loaded onto a single factor. The goodness-of-fit indices (Table 2) supported the adequacy of this model (*χ*^2^ = 1972.918, df = 54, *p* < 0.001; RMR = 0.017; GFI = 0.945; AGFI = 0.921; CFI = 0.951; TLI = 0.940; NFI = 0.950; IFI = 0.951; RMSEA = 0.076). Overall, these indices indicate that the original unidimensional structure (Figure 1) demonstrated an acceptable model fit.

**Table 2:** Goodness-of-fit indices of the PTSD-COVID-19 scale.

Goodness-of-fit index	Value
RMR	0.017
GFI	0.945
AGFI	0.921
CFI	0.951
TLI	0.940
NFI	0.950
IFI	0.951
RMSEA	0.076

RMR, Root Mean Square Residual; GFI, Goodness-of-Fit Index; AGFI, Adjusted Goodness-of-Fit Index; CFI, Comparative Fit Index; TLI, Tucker–Lewis Index; NFI, Normed Fit Index; IFI, Incremental Fit Index; RMSEA, Root Mean Square Error of Approximation.

Finally, the internal consistency of the PTSD-COVID-19 Scale was assessed using Cronbach’s alpha, yielding a value of *α* = 0.92 (95% CI: 0.91–0.92), indicating excellent reliability of the scale.

## Discussion

The present findings support the psychometric usefulness of the PTSD-COVID-19 scale as a brief instrument for screening post-traumatic stress symptoms associated with the early phase of the COVID-19 pandemic in Latin America. The confirmatory factor analysis supported a unidimensional 12-item model with acceptable fit indices (CFI = 0.951, TLI = 0.940, RMSEA = 0.076), and internal consistency was excellent (*α* = 0.92). Rather than suggesting a definitive diagnostic structure, these findings indicate that the scale provides a parsimonious and internally coherent measure of perceived post-traumatic stress symptom burden in this context. For interpretive clarity, Supplementary Table S2 presents the 12 items of the PTSD-COVID-19 scale together with their corresponding conceptual domains, allowing readers to relate the unidimensional CFA findings to the clinical and functional content represented by each item.

These results should be interpreted in relation to the original SPRINT-E validation conducted after the 2010 Chilean earthquake and tsunami. In that study, the scale showed excellent internal consistency (*α* = 0.916), but the two-factor solution showed better fit than the one-factor model. Specifically, the one-factor model yielded CFI = 0.926 and RMSEA = 0.103, whereas the two-factor model yielded CFI = 0.942 and RMSEA = 0.092 (Leiva-Bianchi and Gallardo, 2013). In contrast, the present study retained a unidimensional interpretation with slightly better overall fit than the original one-factor disaster model. This may suggest that, in a large multinational pandemic sample, symptom responses were organized more globally around a common post-traumatic stress dimension than around the more differentiated structure observed after acute natural disaster exposure. At the same time, this comparison should be interpreted cautiously because the original SPRINT-E was developed for a different trauma context, and the present instrument was applied during a prolonged and heterogeneous public health crisis rather than after a sudden catastrophic event.

When compared with more recent psychometric studies published in 2024 and 2025, the present fit indices can be considered acceptable, although less robust than those reported for some multidimensional trauma-related instruments. In Colombian adolescents exposed to adversity, the hybrid PCL-5 model showed RMSEA = 0.0499, CFI = 0.984, TLI = 0.978, and GFI = 0.994 (Alejo and Cuartas Arias, 2025). In a Romanian validation study, the PCL-5 anhedonia and hybrid models showed TLI = 0.95 and 0.95, CFI = 0.93 and 0.94, and RMSEA = 0.06 and 0.06, respectively (Georgescu et al., 2024). In Brazil, the six-factor ITQ model showed CFI = 0.986, TLI = 0.978, and RMSEA = 0.073 (Aprigio et al., 2024). In Norway, the PCL-5 anhedonia and hybrid models showed RMSEA = 0.056 and 0.054, CFI = 0.912 and 0.921, and TLI = 0.892 and 0.899, respectively (Lassen et al., 2025). Similarly, in Indonesia, the seven-factor hybrid PCL-5 model showed excellent fit, with CFI = 0.997, TLI = 0.997, and RMSEA = 0.043 (Ayudia et al., 2025). Taken together, these comparisons suggest that the present scale performs reasonably well as a brief unidimensional screener, although newer multidimensional PTSD instruments may achieve stronger fit when applied to populations with more specific trauma-exposure frameworks and more differentiated latent structures.

A particularly relevant finding concerns item 12, which showed a very low mean (M = 0.202) together with marked positive skewness (2.381) and kurtosis (4.902). This distribution strongly suggests a floor effect and indicates that the item captures a low-frequency but high-severity reaction in the general population. Conceptually, this is coherent if the item reflects fatalistic reactions, self-harm, or suicidal ideation, which would be expected to occur less frequently in a broad community sample during the first months of the pandemic. Thus, item 12 may retain value as a sentinel indicator of severe distress while contributing less to discrimination across the full symptom continuum in nonclinical samples. This interpretation is consistent with the original SPRINT-E expanded version, in which item 12 was treated differently from the other items: it was dichotomous and removed from factor-analytic procedures, being used as a severity criterion rather than as a standard ordinal indicator within the main psychometric model (Leiva-Bianchi and Gallardo, 2013). Therefore, the present distribution of item 12 may not simply reflect poor performance, but rather the fact that it taps a qualitatively more extreme response than the rest of the scale. In this sense, the item may have potential clinical value as a warning flag for high-risk respondents, even if its contribution to total-score discrimination is more limited in community samples.

An additional point concerns the scope of the instrument’s future use. Given its COVID-19-specific framing, the PTSD-COVID-19 scale should not be assumed to transfer directly to other traumatic contexts without adaptation. Its strongest immediate utility lies in research and in early screening of post-traumatic stress symptoms in pandemic-related settings or comparable large-scale health emergencies. In clinical settings, its possible usefulness is more appropriately understood as a first-stage triage or symptom-monitoring tool that may help identify individuals who require more comprehensive evaluation, rather than as a stand-alone diagnostic measure. This interpretation is consistent with the broader literature on brief trauma-related instruments such as the SPRINT, which has shown utility for screening, estimating symptom severity, identifying treatment needs, and monitoring change over time, while formal PTSD diagnosis still requires more comprehensive assessment (Connor and Davidson, 2001). Therefore, any future use of this scale in non-COVID traumatic contexts should involve contextual adaptation, expert review, and renewed psychometric validation before broader application is assumed.

The timing of data collection is also essential for interpretation. Participants were recruited between June and August 2020, during the early phase of the COVID-19 pandemic in Latin America. For that reason, the present findings are better understood as reflecting early pandemic-related post-traumatic symptomatology or acute stress responses than established long-term PTSD trajectories. According to DSM-5-TR, PTSD requires not only exposure to a qualifying traumatic event, but also symptom persistence for more than 1 month and clinically significant distress or functional impairment (American Psychiatric Association, 2022). Because the present study was cross-sectional and based on self-report data, it does not allow confirmation of symptom persistence, full diagnostic criteria, or clinical diagnosis. Therefore, the PTSD-COVID-19 scale should be interpreted as a brief instrument for screening post-traumatic stress symptoms at a specific point in time rather than as a diagnostic tool for chronic PTSD.

## Limitations

This study has several limitations that should be considered when interpreting the findings. First, the study used a non-probabilistic convenience sample, which introduces selection bias and limits strict population-level generalizability. Although the sample was large and geographically diverse, not all Latin American countries were represented, and participants were assessed under the exceptional social conditions of the early pandemic.

Second, the evidence for construct validity was limited mainly to internal structure and internal consistency. No additional validated instruments were administered to examine concurrent, convergent, or divergent validity. Consequently, the present study does not establish how the PTSD-COVID-19 scale performs relative to established trauma-related measures such as the PCL-5, nor does it evaluate divergence from theoretically distinct constructs such as social desirability. This is an important limitation because the PCL-5 is a widely used 20-item self-report measure that can be used for screening and provisional diagnosis of PTSD, whereas the CAPS-5 remains the gold standard structured interview for PTSD assessment (Weathers and W., 2024; Weathers et al., 2018). Future studies should therefore compare the present scale against validated external measures and, when feasible, clinician-administered interviews.

Third, this was a cross-sectional instrumental study conducted during the first months of the COVID-19 pandemic in Latin America. Accordingly, the findings reflect symptom reporting at a specific time point and should not be interpreted as evidence of established clinical PTSD. The retrospective and cross-sectional nature of the study prevents verification of symptom persistence, clinical course, and diagnostic stability over time. Thus, the scale measures perceived post-traumatic stress symptoms in an early and highly specific historical context rather than the longitudinal evolution of the disorder. Future research should address this limitation through longitudinal designs that evaluate symptom persistence, temporal stability, and sensitivity to change.

Fourth, the behavior of item 12 suggests that some contents may function differently in community samples than in highly exposed or clinical trauma samples. Its pronounced skewness and kurtosis indicate that severe fatalistic or self-harm-related responses were infrequent in this population, which may reduce its discriminative utility within the total score. Future studies should examine item-level functioning in greater depth, including item-total correlations, threshold behavior, and the possibility of using this item as a separate clinical flag rather than as a regular severity indicator.

Another limitation is that measurement invariance was not examined across countries or key sociodemographic variables such as sex and age. Therefore, although the scale showed acceptable internal structure and excellent internal consistency in the overall sample, it is not yet possible to determine whether the construct operates equivalently across different national and demographic groups. Future studies should address this issue through multigroup analyses of factorial invariance and should also examine possible differences according to other relevant variables, such as educational level and occupation. These steps would strengthen the cross-cultural interpretability and broader applicability of the instrument in Latin American settings.

In addition, future studies should strengthen the psychometric evidence by evaluating convergent and divergent validity, criterion validity against external standards, test–retest reliability, measurement invariance across countries and sociodemographic groups, and the clinical utility of the instrument in both community and high-risk samples. These steps would provide a more comprehensive understanding of the scale’s role as a brief screening tool in trauma-related contexts.

## Conclusion

In conclusion, the PTSD-COVID-19 scale provides acceptable evidence of internal structure and excellent internal consistency as a brief instrument for screening post-traumatic stress symptoms in Latin American adults during the early phase of the COVID-19 pandemic. Its findings should be interpreted as reflecting symptom burden at a specific historical moment rather than clinically confirmed long-term PTSD. The instrument may be useful in research and in initial symptom screening, but any application beyond the COVID-19 context should require contextual adaptation and renewed psychometric validation.

## Data Availability

The raw data supporting the conclusions of this article will be made available by the authors without undue reservation.
