# Supplementary material for: Psychometric revalidation of the SPRINT-E scale for assessing post-traumatic stress in Latin American populations during the COVID-19 pandemic
Source: Front Psychol. 2026 Apr 8;17:1781414. doi: 10.3389/fpsyg.2026.1781414 (PMC13099782; doi:10.3389/fpsyg.2026.1781414)
Supplement: Supplementary file 1 [file Supplementary_File_1.docx]

**INDICACIONES EN CASTELLANO**

Se considera que un síntoma es intenso si tiene una puntuación de 3 o 4. Con el total de síntomas intensos, se utiliza la “regla de 3/7” que afirma que, si una persona presenta 3 o más síntomas intensos, es muy probable que sufra de TEPT; pero con 7 o más respuestas, la probabilidad de un falso positivo (diagnosticar TEPT cuando una persona no padece esta enfermedad) es muy baja. Ítem 12: Este último ítem no se incluye en la puntuación, pero se incluyó como medida de precaución: cuando se responde afirmativamente a esta pregunta, los profesionales de la salud pueden solicitar una derivación inmediata a servicios psiquiátricos. Instrucciones adaptadas de Leiva-Bianchi MC et al. Validation of the short posttraumatic stress disorder rating interview (SPRINT-E) in a sample of people affected by F-27 Chilean earthquake and tsunami. Anales de psicología. 2013; 29(2): 328-334.

Forma resumida para realizar el análisis con la escala: Si se tiene cuando menos 3 respuestas Regular o Mucho = Muy probable que se sufra de estrés post traumático. Si se tiene cuando menos 7 respuestas Regular o Mucho = Es muy baja la probabilidad de un falso positivo para estrés post traumático. El ítem 12 no se toma en cuenta para esto que se acaba de mencionar.

| **Durante el último mes:** | **N** | **P** | **R** | **M** |
| --- | --- | --- | --- | --- |
| ¿Cuánto le han molestado los recuerdos no deseados o pesadillas de lo que pasó? |  |  |  |  |
| ¿Cuánto esfuerzo ha hecho para evitar pensar o hablar sobre lo sucedido o realizar actos que le recuerden lo sucedido? |  |  |  |  |
| ¿Hasta qué punto ha perdido el placer por las cosas, se mantiene distante de la gente, o le ha sido difícil experimentar sentimientos a consecuencia de lo sucedido? |  |  |  |  |
| ¿Cuánto le han incomodado problemas de sueño, concentración, nerviosismo, irritabilidad o sentirse muy alerta de lo que le rodea a consecuencia de lo sucedido? |  |  |  |  |
| ¿Qué tan desanimado o deprimido se tiene sentido a consecuencia de lo sucedido? |  |  |  |  |
| ¿Considera que su habilidad para manejar otras situaciones o eventos estresantes se ha visto dañada? |  |  |  |  |
| ¿Considera que sus reacciones interfieren con el cuidado de su salud física? Por ejemplo, ¿se alimenta poco, no descansa suficiente, fuma más, o se ha dado cuenta que ha incrementado el uso de alcohol y otras sustancias? |  |  |  |  |
| ¿Qué tan estresado o incómodo se siente con respecto a sus reacciones? |  |  |  |  |
| ¿Qué tanto han interferido sus reacciones con su habilidad para trabajar o llevar a cabo actividades diarias, como labores del hogar o académicas? |  |  |  |  |
| Dadas sus reacciones ¿qué tan afectada se han visto sus relaciones familiares o de amistad?, ¿qué tanto han interferido en sus actividades sociales, recreativas o comunitarias? |  |  |  |  |
| ¿Qué tan preocupado se ha sentido acerca de su habilidad para vencer los problemas que podría enfrentar sin mayor asistencia? |  |  |  |  |
| ¿Hay alguna posibilidad de que usted tenga deseos de herirse o suicidarse? | Si ( ) No ( ) | | | |

N: Nunca; P: Poco; R: Regular; M: Mucho.

**INSTRUCTIONS IN ENGLISH**

A symptom is considered "intense" if it receives a score of 3 or 4. Based on the total number of intense symptoms, the "3/7 rule" is applied: if a person presents with 3 or more intense symptoms, they probably suffer from PTSD. However, with 7 or more such responses, the likelihood of a false positive (diagnosing PTSD when the person does not actually suffer from the disorder) is very low. Item 12: This final item is not included in the scoring, but it was included as a precautionary measure: if this question is answered affirmatively, health professionals may request an immediate referral to psychiatric services. Instructions adapted fromLeiva-Bianchi MC et al. Validation of the short posttraumatic stress disorder rating interview (SPRINT-E) in a sample of people affected by F-27 Chilean earthquake and tsunami. Anales de psicología. 2013; 29(2): 328-334.

Summary guide for analyzing the scale results: If there are at least 3 responses marked "Regular" or "Much" = It is highly probable that the individual suffers from post-traumatic stress. If there are at least 7 responses marked "Regular" or "Much" = The likelihood of a false positive for post-traumatic stress is very low. Item 12 is not considered for the criteria just mentioned.

| **During the last month:** | **N** | **R** | **S** | **O** |
| --- | --- | --- | --- | --- |
| To what extent have you been troubled by unwanted memories or nightmares of what happened? |  |  |  |  |
| How much effort have you made to avoid thinking or talking about what happened, or to avoid doing things that remind you of it? |  |  |  |  |
| To what extent have you lost interest in things, felt distant from people, or found it difficult to experience feelings because of what happened? |  |  |  |  |
| To what extent have you been distressed by problems with sleep, concentration, nervousness, irritability, or feeling overly alert to your surroundings because of what happened? |  |  |  |  |
| How discouraged or depressed have you felt because of what happened? |  |  |  |  |
| Do you feel that your ability to handle other stressful situations or events has been impaired? |  |  |  |  |
| Do you feel that your reactions interfere with your ability to take care of your physical health? For example, are you eating too little, not getting enough rest, smoking more, or have you noticed an increase in your use of alcohol or other substances? |  |  |  |  |
| How stressed or uncomfortable do you feel regarding your reactions? |  |  |  |  |
| To what extent have your reactions interfered with your ability to work or carry out daily activities, such as household chores or academic tasks? |  |  |  |  |
| Given your reactions, how have your family relationships or friendships been affected? To what extent have they interfered with your social, recreational, or community activities? |  |  |  |  |
| How worried have you felt about your ability to overcome the problems you might face without additional assistance? |  |  |  |  |
| Is there any possibility that you have thoughts of harming yourself or committing suicide? | Yes ( ) No ( ) | | | |

N: Never; R: Rarely; S: Sometimes; O: Often
