# Supplementary material for: Psychometric revalidation of the SPRINT-E scale for assessing post-traumatic stress in Latin American populations during the COVID-19 pandemic
Source: Front Psychol. 2026 Apr 8;17:1781414. doi: 10.3389/fpsyg.2026.1781414 (PMC13099782; doi:10.3389/fpsyg.2026.1781414)
Supplement: Supplementary file 2 [file Table_1.docx]

**Supplementary Table S1. Conceptual content of the PTSD-COVID-19 scale**

|  | | | Ítem1 | Ítem2 | Ítem3 | Ítem4 | Ítem5 | Ítem6 | Ítem7 | Ítem8 | Ítem9 | Ítem10 | Ítem11 | Ítem12 |
| --- | --- | --- | --- | --- | --- | --- | --- | --- | --- | --- | --- | --- | --- | --- |
| Bolivia | Masculino | Media | 0.44 | 0.47 | 0.60 | 0.75 | 0.50 | 0.43 | 0.50 | 0.61 | 0.57 | 0.59 | 0.49 | 0.23 |
|  |  | SD | 0.625 | 0.655 | 0.700 | 0.826 | 0.682 | 0.691 | 0.732 | 0.699 | 0.764 | 0.738 | 0.719 | 0.549 |
|  | Femenino | Media | 0.69 | 0.78 | 0.79 | 1.06 | 0.90 | 0.71 | 0.69 | 1.02 | 0.97 | 0.97 | 0.89 | 0.22 |
|  |  | SD | 0.729 | 0.761 | 0.764 | 0.809 | 0.718 | 0.731 | 0.799 | 0.741 | 0.783 | 0.780 | 0.746 | 0.510 |
| Chile | Masculino | Media | 0.42 | 0.47 | 0.56 | 0.76 | 0.64 | 0.54 | 0.59 | 0.58 | 0.58 | 0.68 | 0.53 | 0.13 |
|  |  | SD | 0.628 | 0.640 | 0.758 | 0.785 | 0.698 | 0.698 | 0.760 | 0.688 | 0.713 | 0.683 | 0.660 | 0.420 |
|  | Femenino | Media | 0.67 | 0.74 | 0.79 | 1.18 | 0.99 | 0.80 | 0.93 | 1.00 | 0.93 | 0.92 | 0.79 | 0.17 |
|  |  | SD | 0.711 | 0.765 | 0.815 | 0.809 | 0.737 | 0.794 | 0.856 | 0.813 | 0.840 | 0.755 | 0.770 | 0.450 |
| Colombia | Masculino | Media | 0.32 | 0.36 | 0.64 | 0.64 | 0.59 | 0.27 | 0.41 | 0.50 | 0.55 | 0.68 | 0.45 | 0.14 |
|  |  | SD | 0.646 | 0.727 | 0.902 | 0.727 | 0.796 | 0.550 | 0.590 | 0.673 | 0.800 | 0.839 | 0.671 | 0.468 |
|  | Femenino | Media | 0.46 | 0.74 | 0.83 | 1.09 | 0.89 | 0.69 | 0.74 | 0.97 | 0.94 | 0.94 | 0.80 | 0.34 |
|  |  | SD | 0.561 | 0.780 | 0.747 | 0.612 | 0.758 | 0.631 | 0.780 | 0.785 | 0.684 | 0.725 | 0.719 | 0.591 |
| Costa Rica | Masculino | Media | 0.35 | 0.33 | 0.58 | 0.45 | 0.50 | 0.33 | 0.48 | 0.48 | 0.58 | 0.60 | 0.38 | 0.15 |
|  |  | SD | 0.700 | 0.656 | 0.747 | 0.714 | 0.751 | 0.616 | 0.751 | 0.599 | 0.781 | 0.744 | 0.667 | 0.483 |
|  | Femenino | Media | 0.51 | 0.57 | 0.57 | 0.83 | 0.49 | 0.44 | 0.53 | 0.53 | 0.43 | 0.68 | 0.48 | 0.15 |
|  |  | SD | 0.742 | 0.738 | 0.756 | 0.760 | 0.645 | 0.620 | 0.741 | 0.704 | 0.640 | 0.791 | 0.665 | 0.485 |
| Ecuador | Masculino | Media | 0.37 | 0.56 | 0.62 | 0.51 | 0.65 | 0.43 | 0.48 | 0.64 | 0.56 | 0.59 | 0.62 | 0.27 |
|  |  | SD | 0.588 | 0.645 | 0.704 | 0.675 | 0.699 | 0.644 | 0.688 | 0.739 | 0.726 | 0.710 | 0.704 | 0.503 |
|  | Femenino | Media | 0.70 | 0.83 | 0.76 | 0.94 | 0.87 | 0.62 | 0.65 | 0.84 | 0.76 | 0.78 | 0.69 | 0.30 |
|  |  | SD | 0.720 | 0.757 | 0.775 | 0.782 | 0.731 | 0.710 | 0.752 | 0.754 | 0.769 | 0.779 | 0.752 | 0.580 |
| El Salvador | Masculino | Media | 0.28 | 0.53 | 0.48 | 0.93 | 0.68 | 0.40 | 0.58 | 0.63 | 0.55 | 0.63 | 0.35 | 0.08 |
|  |  | SD | 0.506 | 0.679 | 0.640 | 0.764 | 0.616 | 0.672 | 0.813 | 0.740 | 0.677 | 0.667 | 0.662 | 0.267 |
|  | Femenino | Media | 0.63 | 0.76 | 0.75 | 1.25 | 0.91 | 0.72 | 0.93 | 0.99 | 0.94 | 0.78 | 0.73 | 0.09 |
|  |  | SD | 0.714 | 0.761 | 0.766 | 0.785 | 0.733 | 0.670 | 0.876 | 0.707 | 0.776 | 0.670 | 0.687 | 0.336 |
| Guatemala | Masculino | Media | 0.20 | 0.20 | 0.40 | 0.40 | 0.30 | 0.20 | 0.30 | 0.20 | 0.30 | 0.40 | 0.10 | 0.10 |
|  |  | SD | 0.422 | 0.422 | 0.699 | 0.699 | 0.483 | 0.422 | 0.675 | 0.422 | 0.483 | 0.699 | 0.316 | 0.316 |
|  | Femenino | Media | 0.36 | 0.57 | 0.64 | 0.86 | 0.64 | 0.43 | 0.64 | 0.79 | 0.86 | 0.79 | 0.64 | 0.14 |
|  |  | SD | 0.633 | 0.756 | 0.745 | 0.770 | 0.633 | 0.646 | 0.842 | 0.802 | 0.864 | 0.802 | 0.633 | 0.363 |
| Honduras | Masculino | Media | 0.56 | 0.38 | 0.56 | 0.56 | 0.56 | 0.38 | 0.50 | 0.63 | 0.38 | 0.31 | 0.44 | 0.19 |
|  |  | SD | 0.727 | 0.619 | 0.629 | 0.629 | 0.629 | 0.500 | 0.632 | 0.619 | 0.619 | 0.479 | 0.512 | 0.403 |
|  | Femenino | Media | 0.51 | 0.67 | 0.62 | 0.95 | 0.74 | 0.41 | 0.59 | 0.67 | 0.56 | 0.56 | 0.59 | 0.15 |
|  |  | SD | 0.644 | 0.701 | 0.747 | 0.793 | 0.785 | 0.715 | 0.785 | 0.737 | 0.680 | 0.552 | 0.751 | 0.366 |
| México | Masculino | Media | 0.29 | 0.39 | 0.41 | 0.53 | 0.50 | 0.35 | 0.47 | 0.50 | 0.59 | 0.54 | 0.40 | 0.17 |
|  |  | SD | 0.551 | 0.564 | 0.568 | 0.653 | 0.641 | 0.555 | 0.665 | 0.641 | 0.634 | 0.627 | 0.594 | 0.456 |
|  | Femenino | Media | 0.57 | 0.65 | 0.64 | 0.98 | 0.83 | 0.63 | 0.74 | 0.85 | 0.80 | 0.71 | 0.67 | 0.21 |
|  |  | SD | 0.735 | 0.723 | 0.735 | 0.850 | 0.755 | 0.709 | 0.786 | 0.790 | 0.768 | 0.730 | 0.740 | 0.491 |
| Panamá | Masculino | Media | 0.34 | 0.30 | 0.43 | 0.61 | 0.62 | 0.44 | 0.46 | 0.57 | 0.59 | 0.48 | 0.31 | 0.16 |
|  |  | SD | 0.602 | 0.641 | 0.618 | 0.781 | 0.756 | 0.620 | 0.697 | 0.784 | 0.761 | 0.698 | 0.564 | 0.454 |
|  | Femenino | Media | 0.38 | 0.57 | 0.38 | 0.69 | 0.56 | 0.31 | 0.31 | 0.54 | 0.41 | 0.64 | 0.46 | 0.07 |
|  |  | SD | 0.582 | 0.763 | 0.637 | 0.786 | 0.696 | 0.564 | 0.620 | 0.721 | 0.642 | 0.775 | 0.673 | 0.309 |
| Paraguay | Masculino | Media | 0.49 | 0.52 | 0.57 | 0.64 | 0.55 | 0.41 | 0.45 | 0.53 | 0.60 | 0.64 | 0.47 | 0.18 |
|  |  | SD | 0.722 | 0.673 | 0.703 | 0.761 | 0.663 | 0.615 | 0.680 | 0.698 | 0.706 | 0.761 | 0.656 | 0.476 |
|  | Femenino | Media | 0.64 | 0.67 | 0.70 | 0.94 | 0.85 | 0.60 | 0.65 | 0.85 | 0.79 | 0.83 | 0.74 | 0.21 |
|  |  | SD | 0.710 | 0.726 | 0.725 | 0.822 | 0.792 | 0.712 | 0.788 | 0.766 | 0.762 | 0.793 | 0.740 | 0.505 |
| Perú | Masculino | Media | 0.46 | 0.52 | 0.53 | 0.63 | 0.54 | 0.47 | 0.45 | 0.63 | 0.58 | 0.58 | 0.50 | 0.19 |
|  |  | SD | 0.627 | 0.639 | 0.669 | 0.715 | 0.658 | 0.633 | 0.652 | 0.681 | 0.686 | 0.689 | 0.628 | 0.462 |
|  | Femenino | Media | 0.68 | 0.74 | 0.76 | 0.97 | 0.87 | 0.70 | 0.65 | 0.92 | 0.83 | 0.82 | 0.78 | 0.23 |
|  |  | SD | 0.710 | 0.720 | 0.734 | 0.764 | 0.707 | 0.701 | 0.757 | 0.734 | 0.735 | 0.723 | 0.717 | 0.500 |
