# Supplementary material for: Psychometric revalidation of the SPRINT-E scale for assessing post-traumatic stress in Latin American populations during the COVID-19 pandemic
Source: Front Psychol. 2026 Apr 8;17:1781414. doi: 10.3389/fpsyg.2026.1781414 (PMC13099782; doi:10.3389/fpsyg.2026.1781414)
Supplement: Supplementary file 3 [file Table_2.docx]

**Supplementary Table S2. Conceptual content of the PTSD-COVID-19 scale**

| **Item** | **Conceptual content / domain** |
| --- | --- |
| 1 | Intrusive memories |
| 2 | Disturbing dreams or trauma-related sleep experiences |
| 3 | Re-experiencing of the event |
| 4 | Psychological distress associated with reminders |
| 5 | Avoidance or emotional withdrawal |
| 6 | Reduced stress tolerance or difficulty coping |
| 7 | Interference with self-care and health behaviors |
| 8 | Distress caused by one’s own reactions |
| 9 | Difficulty solving daily problems or functional coping |
| 10 | Interference in family and social relationships |
| 11 | Interference in work, household, or academic functioning |
| 12 | Fatalistic response or self-harm/suicidal ideation indicator |
